# Wise Pattern Mammoplasty: The Ideal Option for Giant Fibroadenoma Resection

**DOI:** 10.7759/cureus.79677

**Published:** 2025-02-26

**Authors:** Suhair Al Saad, Hamdi Al Shenawi, Noor Al Shenawi, Herminia AlSaffar, Fedaa Al Sindi

**Affiliations:** 1 Breast Surgery, Dr. Suhair Al Saad Medical Centre, Manama, BHR; 2 Surgery, Arabian Gulf University, Manama, BHR; 3 Radiology, Al Khaleej Polyclinics, Manama, BHR; 4 Department of Pathology, Blood Bank and Laboratory Medicine, King Hamad University Hospital, Muharraq, BHR

**Keywords:** bahrain, breast, giant fibroadenoma, surgery, wise pattern mammoplasty

## Abstract

Fibroadenoma is a benign tumor of the breast and mainly affects women in early adolescence. Giant fibroadenomas are less common and can be misdiagnosed with breast hypertrophy, pseudo-angiomatous stromal hyperplasia, or phyllodes. Hence, a pre-operative biopsy is mandatory for diagnosis and surgical planning. Wise pattern reduction mammoplasty is ideal for the resection of fibroadenomas. Here, we present a case of a 23-year-old woman who presented with a breast lump diagnosed as giant fibroadenoma. She underwent complete tumor resection using cosmetic wise pattern mammoplasty that is considered one of the best options to preserve the breast shape with negligible deformity.

## Introduction

Fibroadenomas are the most common, benign breast tumors affecting women, typically occurring in their 20s and 30s. Fibroadenomas are made up of both glandular tissue and connective tissue. It's a gradual disease process as it tends to shrink with time and may calcify after menopause [1]. Fibroadenomas are typically painless, round, well-defined lumps, freely mobile, and felt as either firm or rubbery in consistency. Some are small, non-palpable, and only diagnosed by breast imaging using ultrasound or mammography [1,2].

To confirm the diagnosis, a breast biopsy is needed, before planning treatment. Small fibroadenomas in young patients may not require surgical excision as they may disappear or stay stable in size on follow-up clinical examination or breast imaging [1]. Most fibroadenomas are simple fibroadenomas. A less common subtype is complex fibroadenomas, which have a characteristic histopathology and occur at an older age. The histopathology includes cysts, sclerosing adenosis, epithelial calcifications, or papillary apocrine metaplasia. Dupont et al. found an increased risk of malignant transformation, but other studies did not report the same [1-6].

## Case presentation

A 23-year-old young Bahraini woman, who was a final year college student, presented with a left breast lump of six-month duration. The breast lump was rapidly growing in size with no associated pain, nipple discharge, or skin redness. There was no fever or history of trauma. She had her menarche at 15 years of age; her periods were regular with no significant past medical or surgical history. She had no family history of breast cancer or any other malignancy. She was 45 kg in weight and 160 cm in height. A breast examination showed a huge left breast lump, almost quadruple the size of her right breast. The skin overlying the left breast was thinned out with stretch marks and dilated veins over the lateral aspect. The left nipple-areola complex (NAC) was pushed medially by a huge soft palpable mass occupying the lateral half, mainly, the upper outer quadrant of her breast (around 15x10 cm^2^). The right breast was free of any lumps or abnormalities. Both axillae were free of any masses or lymphadenopathy.

**Figure 1:**
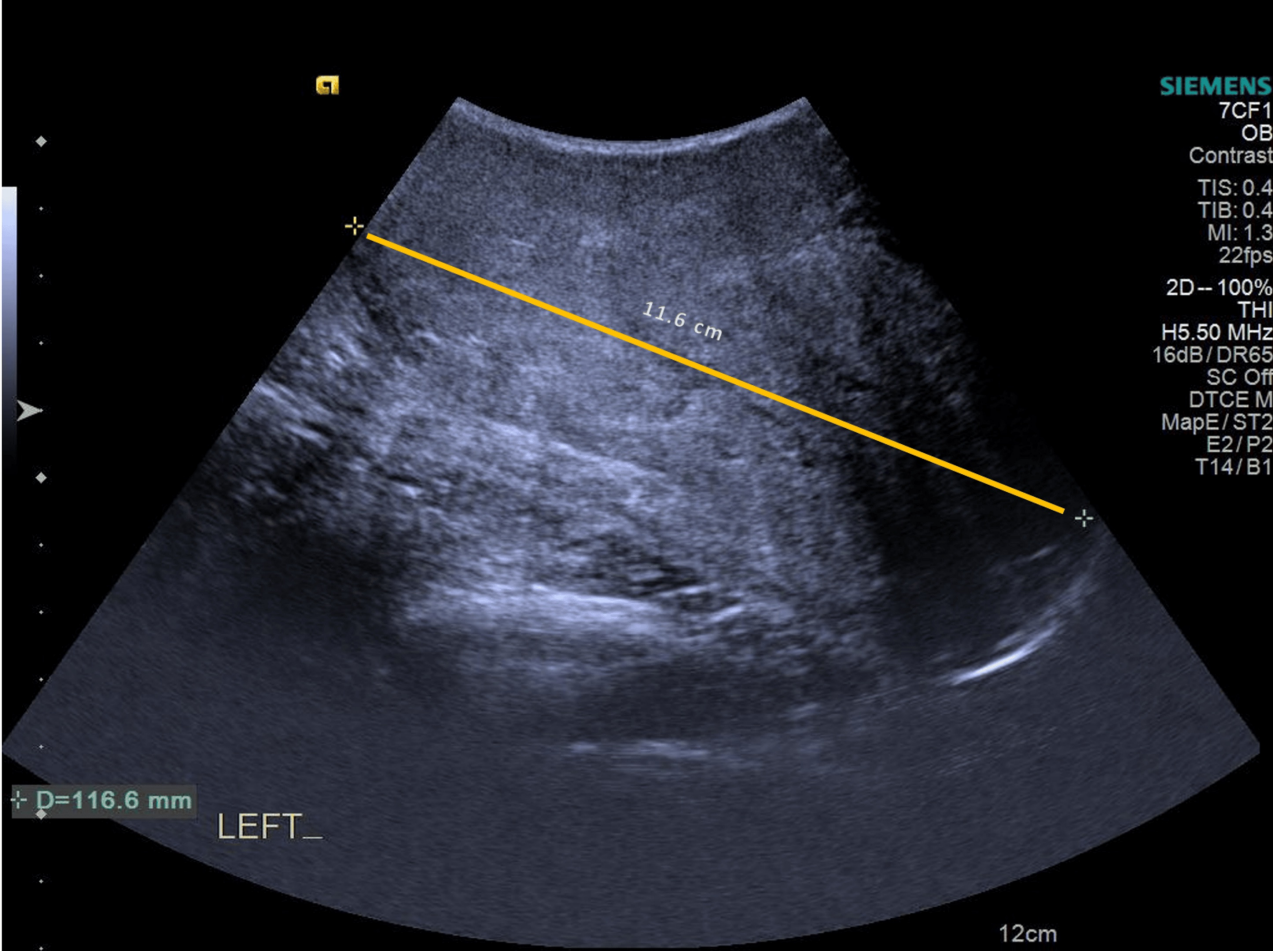
A large field-of-view ultrasound image demonstrating a 11.6-cm heterogeneous hypoechoic solid mass in the left breast.

**Figure 2:**
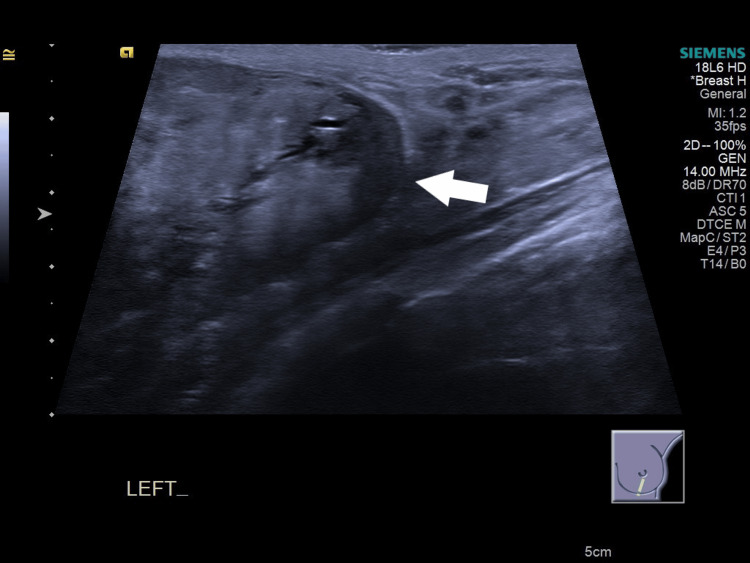
A high-resolution ultrasound image showing part of the lower margin of the left breast mass. The lesion is heterogeneous and predominantly solid with a few small cystic areas. The margins of the mass are smooth (arrow).

She had a breast ultrasound that showed a heterogeneous hypoechoic solid mass in the left breast and internal cystic areas. The tumor margins were smooth, and its size was around 12 cm. Phyllodes tumor could not be excluded (Figures 1, 2).

**Figure 3:**
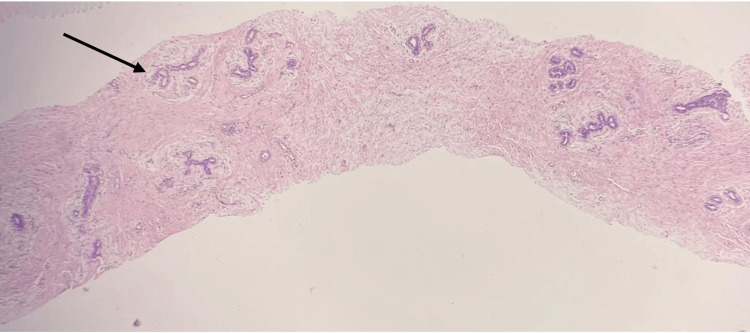
The core biopsy showing a nodular stromoepitheilal tumor with a pericanalicular growth pattern (H&E-stained, medium-power field).

**Figure 4:**
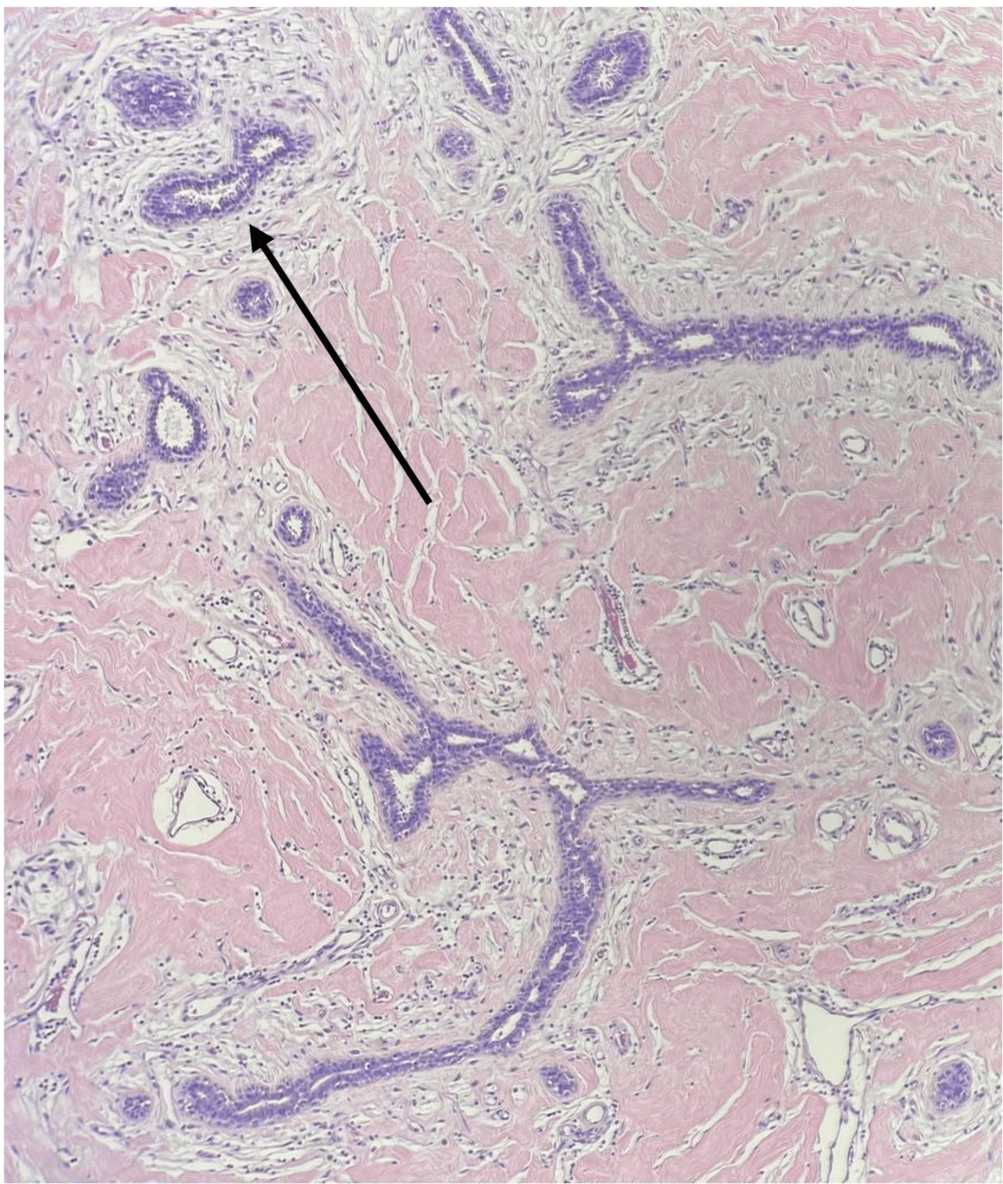
A magnified picture of the core biopsy showing a nodular stromoepithelial tumor with an intracanalicular growth pattern (H&E-stained, high-power field).

A core biopsy was done. The histopathology report revealed a benign nodular stromoepithelial tumor with mixed peri- and intracanalicular growth patterns, consistent with a fibroadenoma, classified as B2 (Figures 3, 4).

We planned to perform a cosmetic resection of the fibroadenoma. Surgical options were discussed with the patient and her parents. The decision was to complete the excision of the left breast mass through a wise pattern therapeutic reduction mammoplasty incision (inverted T). This decision was reached because the outcomes of this surgical approach have better cosmetic results as it maintains the breast shape and the nipple-areola complex is centralized.

**Figure 5:**
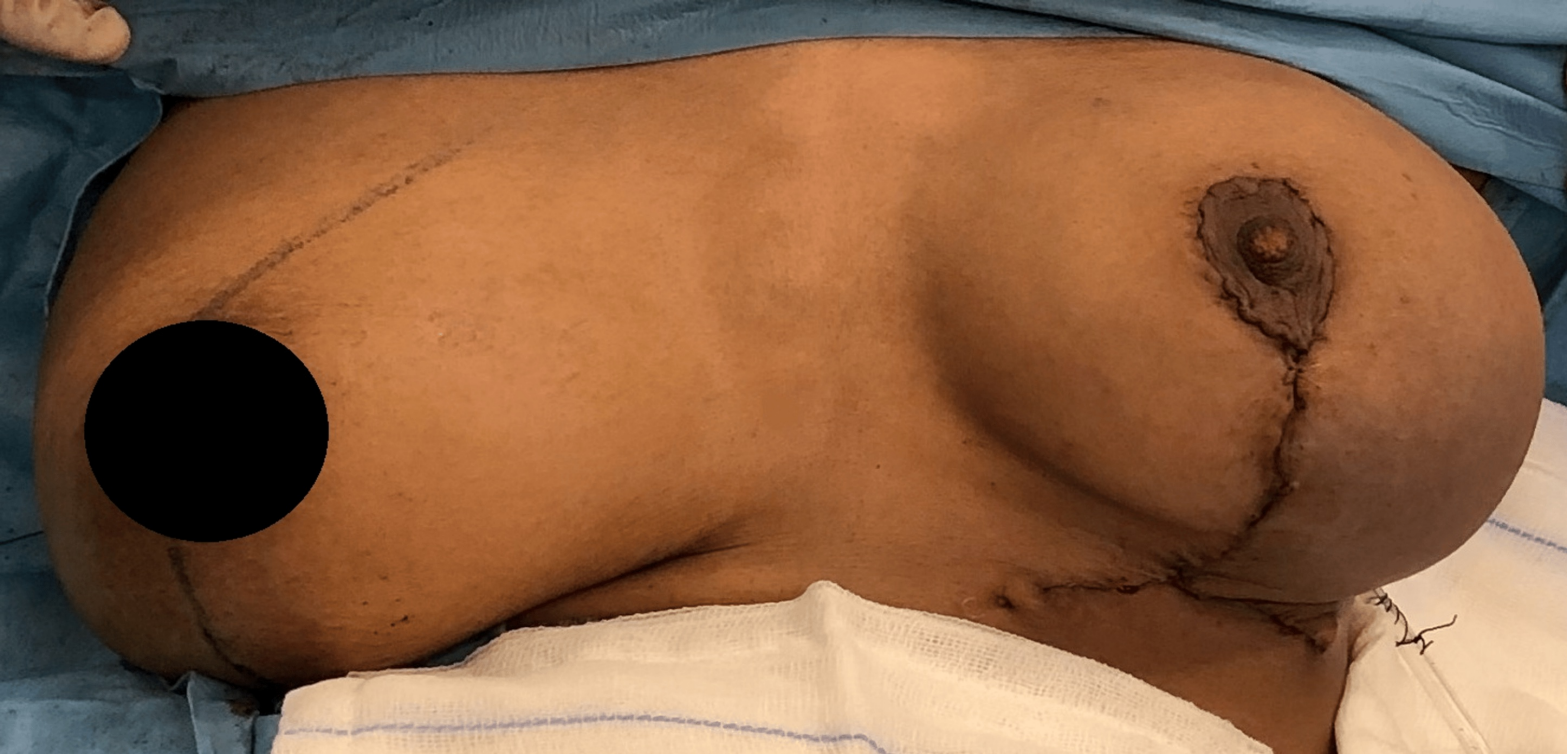
Postoperative image, at the end of the surgery (left breast therapeutic reduction mammoplasty through wise pattern incision).

The wise pattern approach is usually done while marking the patient in a standing position, to mark the new position of the new NAC and the two vertical limbs from the new NAC and the two horizontal extensions to the inferior mammary line. Through these incisions, the tumor is resected and the remaining breast tissue is mobilized to close the cavity. This is followed by the repositioning of the NAC and the closure of the medial and lateral flaps. The operation was uneventful, and the remaining normal compressed breast tissue expanded and filled the space of the resected mass (Figure 5).

**Figure 6:**
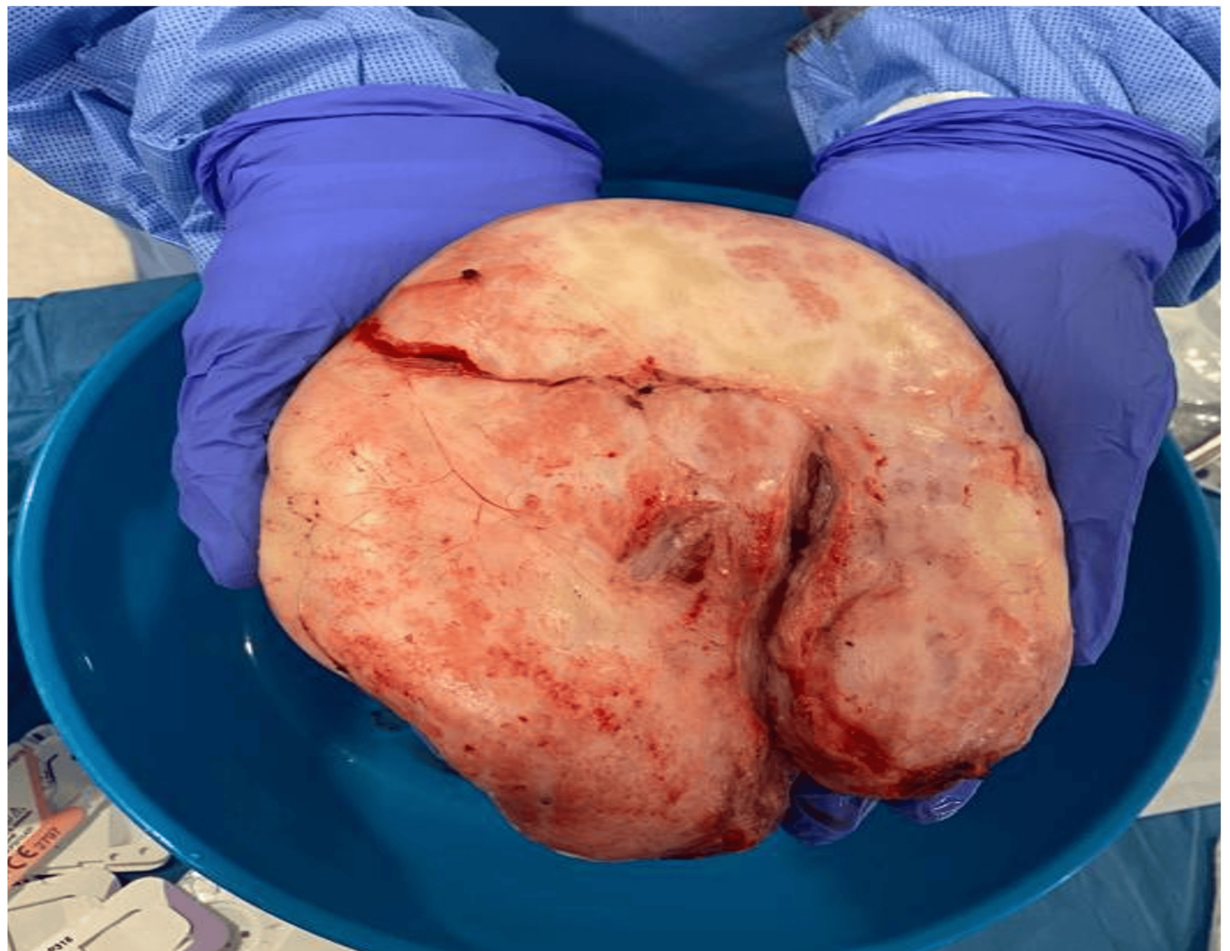
The resected mass from the left breast (weight, 550 gm).

The histopathology of the excisional biopsy showed grossly that the tumor size was 150x140x48 mm^3^. The tumor was rubbery and weighed 550 gm (Figure 6).

**Figure 7:**
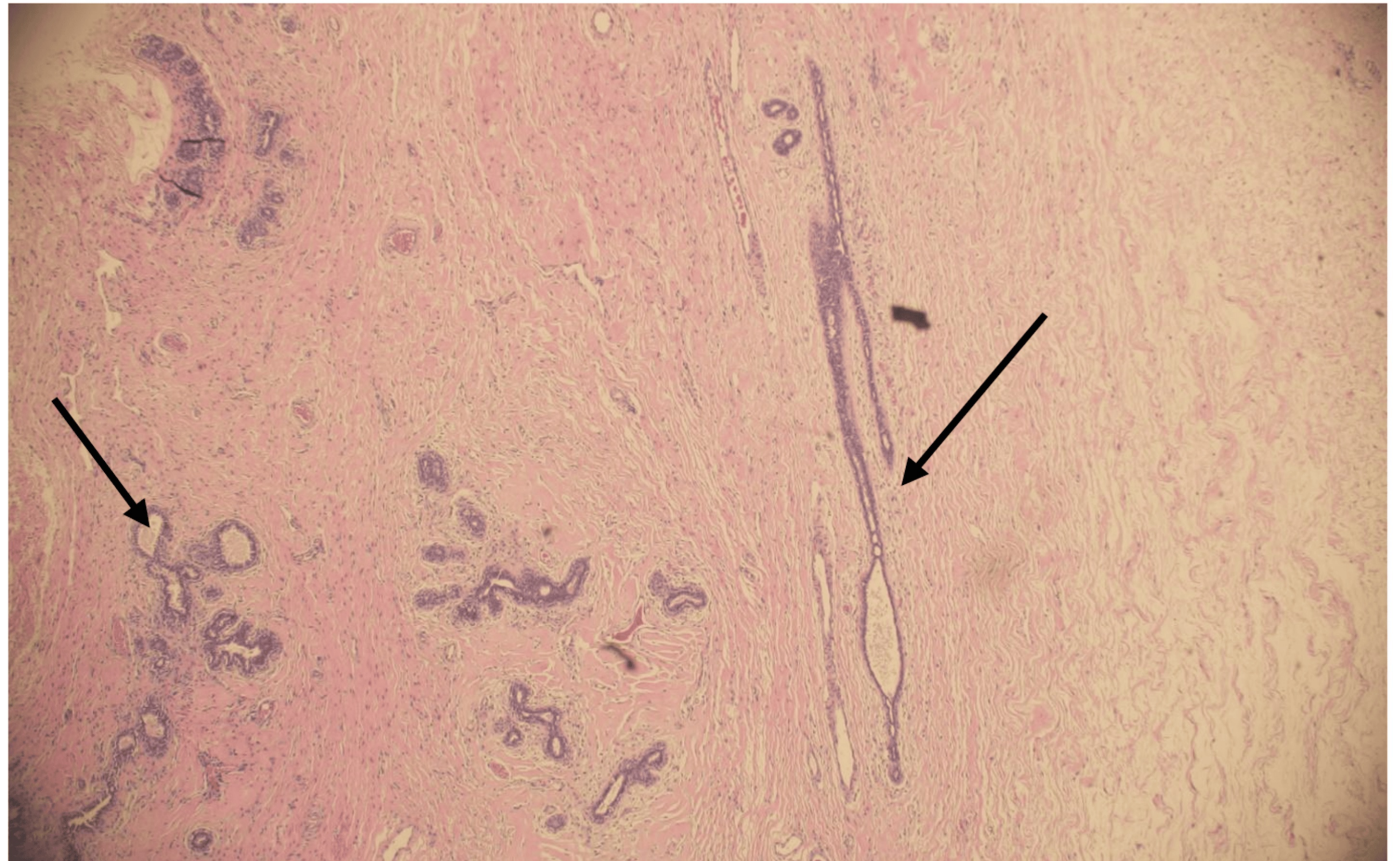
The histopathology of the excised specimen showed mixed intracanalicular (left arrow) and pericanalicular (right arrow) growth patterns. The stroma was hypocellular and formed by variable fibrocollagenous tissue, with stromal myxoid changes (H&E stain).

Microscopically, the tumor was formed by benign mixed stromoepithelial tissue elements, but with a relative predominance of the stromal fibrocollagenous component. Variable myxoid changes were also seen. Peri- and intracanlicular patterns formed the epithelial component, but it was largely formed by a pericanalicular pattern in which no significant hyperplastic changes were found. A thorough examination revealed no significant stromal hypercellularity, cellular atypia, or mitoses (Figure 7).

**Figure 8:**
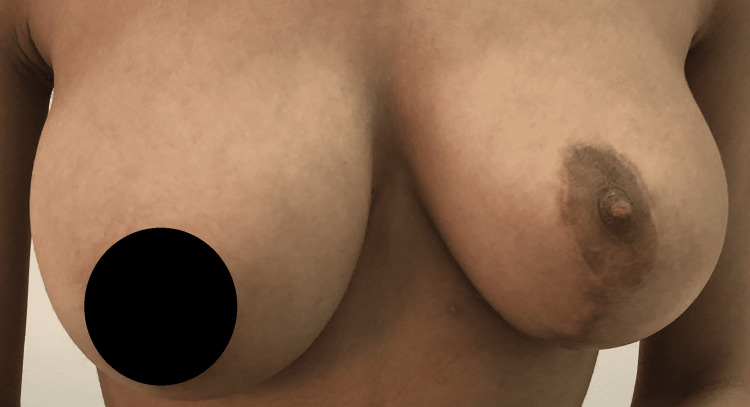
The one-year postoperative image showing the left breast with good cosmetic results. The right breast is ptotic and bigger than the operated side.

Histopathology findings confirmed the diagnosis of a giant fibroadenoma. Postoperatively, she developed a minor superficial wound infection at the junction of the T scar that improved on local dressing. A one-year follow-up showed no local recurrence of the fibroadenoma. The left breast size improved but was slightly smaller than the right (Figure 8).

The patient was given the option of left breast lipofilling and right breast reduction mammoplasty; however, she expressed disinterest and refused.

## Discussion

The giant fibroadenoma is rare and defined as a fibroadenoma that replaces 80% of the breast tissue, i.e., greater than 5 cm in size, or with a weight of 500 g and above [7]. It accounts for only 0.5%-2% of all fibroadenomas as reported by several studies [1,8-10]. As reported by another study, in the adolescent population, it accounts for approximately 1%-8% of all breast lesions [11]. Giant fibroadenoma occurs usually during adolescence, with its peak incidence mostly between the ages of 10 and 18 years [1,12]. If diagnosed at this age, it is considered juvenile giant fibroadenoma [12]. One unusual case was reported in Japan in a 39-year-old woman who presented with breast ulceration and bleeding [8]. Most cases are reported in Asian and African American women [1]. Although they present unilaterally, bilateral cases are also reported [1,10,12]. They are frequently seen during puberty, pregnancy, lactation, and in response to oral contraceptive pills [1,8]. The etiology is unknown but could be related to overstimulation by excessive endogenous or exogenous estrogen, in the form of oral contraceptives or others. It could also be due to the hypersensitivity of mammary gland tissue to estrogen or deficiency of progesterone [1,10,12].

A rapid increase in the breast size can lead to anxiety in both the patients and their parents and can have a psychological and emotional impact that can affect a patient's self-image and relationship with peers [4,12-14]. This was also seen in our case preoperatively, followed by a dramatic psychological improvement after the surgery.

In a review analysis of 52 studies on giant fibroadenomas, 153 patients were studied. Most of the patients (92.9%) did not have any associated condition. The remaining 5.2% were associated with conditions like neuroblastoma, end-stage renal disease, and generalized body hemihypertrophy of an unknown cause. Other conditions such as Beckwith-Wiedemann syndrome, Turner syndrome, severe scoliosis, congenital tubular breast disease, and androgen insensitivity syndrome were also present in some cases. This study could not explain any direct relation between giant fibroadenomas and these conditions [7].

In the literature, the differential diagnosis of a large painless breast lump in a young woman, as seen in our case, includes juvenile macromastia (breast physiologic hypertrophy), lipoma, pseudoangiomatous stromal hyperplasia (PASH), hamartoma, and lastly, phyllodes tumor and breast cancer, which are the most serious [7,10]. We reported in 2009, a case of PASH in a 28-year-old married woman who presented with a painless, rapidly growing mass of one-year duration in her breast [15]. PASH is a benign disease. The mass was big (400 g in weight and 10x10x6 cm in size), and both ultrasound and fine-needle aspiration cytology (FNAC) were inconclusive. She underwent wide local excision with a free margin, and there was no local recurrence on follow-up [15].

It is difficult to differentiate phyllodes tumors from giant fibroadenomas by breast imaging as breast ultrasound, mammogram or MRI cannot distinguish between them. Some articles suggest that mammograms and FNAC can confirm the diagnosis, but there is not enough data to support this [7]. In the PASH case we reported in 2009, it was also difficult to differentiate it from phyllodes by FNAC [15]. In 2011, we also reported a case of a 53-year-old woman with a malignant phyllodes tumor who had the same presentation of a rapidly growing mass in her breast and was difficult to diagnose by mammogram, breast ultrasound, and FNAC. Even a core biopsy could not differentiate if the phyllodes tumor was benign, borderline, or malignant. The tumor size was big and replaced her breast tissue (size: 15x15 cm^2^), which mostly raised the possibility of malignancy [16]. This patient underwent mastectomy and the histopathology confirmed the diagnosis. She died of disseminated disease a few months later.

A core biopsy of the lump is still the best tool for diagnosis [1]. A huge giant fibroadenoma, similar to that in our patient, may present with skin dimpling, superficial breast vein dilation, and peau d’orange resembling phyllodes or cancer [16].

A preoperative core biopsy and histopathological confirmation allow for proper planning for further management and prognosis. In our case, breast ultrasound was not diagnostic. The core biopsy confirmed the diagnosis of a giant fibroadenoma and allowed us to plan surgery. The histopathology of a giant juvenile fibroadenoma is similar to its classical adulthood counterpart. Both show mixed pericanalicular and intracanalicular growth patterns, and variable stromal hyalinization with epithelial hyperplasia [13]. Our case showed both intra- and pericanalicular growth patterns.

The treatment of fibroadenomas ranges from no treatment with continuous clinical and ultrasonic monitoring every six months to surgical resection depending on the size of the lump, patient's age, patient's preference, and the presence of a positive family history of breast cancer. This wide range of treatment options is because of the reported incidence of complete regression in 10%-59% of cases [16]. This regression could be secondary to infarction, calcification, and hyalinization [1,7].

Nowadays, new options are available for removing small fibroadenomas under local anesthesia by cryoablation or ultrasound-guided therapeutic excisional vacuum-assisted biopsy [17]. No guidelines exist for the best surgical resection option for such giant fibroadenomas [7,16]. Surgical management ranges from simple enucleation to mastectomy with or without reconstruction depending on the age of the patient, degree of breast development, and patient preference [7,16]. In our opinion, the best surgical resection option is preoperative planning with simple cosmetic scars, if possible, to avoid deformity.

Chang et al. recommended three principles in breast reconstruction. The first principle is excision of the lesion and preservation of all the normal breast tissue. The second principle is adjusting and modifying the skin envelope by a well-planned cosmetic scar, and the third principle is positioning the nipple-areola complex aiming for symmetrisation with the opposite breast [3]. In our case, we followed these principles. The mass was resected and the normal breast tissue, which was compressed by the giant tumor, was mobilized. The nipple-areola complex was positioned in the pre-planned position and medial and lateral flaps were closed with minimal skin resection. Wise pattern mammoplasty allowed for complete resection of the mass, centralization of the NAC and minimal skin loss. Postoperatively, the inverted T scar healed well; this prevented deformity and dimpling, but left behind a cosmetic scar.

Mastectomy is one of the modalities used in the treatment of giant fibroadenomas, but is usually reserved for recurrent cases [3,7]. It is also reported that the recurrence rate within the first five years of follow-up after surgery is about 33% [1]. Another systematic review study showed a lower recurrence rate, and only 3.9% of the cases needed re-excision [7].

## Conclusions

Giant fibroadenoma is a rare condition that can grow rapidly in a short period leading to anxiety in patients, and diagnostic difficulties in distinguishing them from other benign and serious conditions like phyllodes. Preoperative diagnosis with the help of a core biopsy allows for proper surgical planning and cosmetic resection in such cases. Wise pattern mammoplasty is considered one of the best options to preserve the breast shape with negligible deformity.
